# New York Lunatic Asylum

**Published:** 1854-04

**Authors:** 


### NEW YORK LUNATIC ASYLUM.

We have before us the eleventh "Annual Report of the Managers of the State Lunatic Asylum of the State of New York," which shows that the results of the Institution have been no less beneficial than in any previous year of its existence. But satisfactory as were the general results it appears that sixty applications for admission were refused for want of room for their accommodation—a story repeated, from year to year, by most of the Lunatic Asylums of the States, testifying to the alarming prevalence of insanity in our country. This report goes on to say:—

"The whole number admitted since the Asylum was opened, on the 16th of January, 1843, is 3,923; of whom 1,625 have been discharged recovered, 55 much improved, 598 improved, 753 unimproved, and 446 have died.

"The number admitted during the year was 424; giving a total of 849 under treatment in the course of the year. Of this number, 169 were discharged recovered, 21 much improved, 45 improved, 129 unimproved, and 39 have died; leaving 446 remaining at the end of the year. We refer to the report of the superintendent for a full statement of the operations and results of the Asylum during the year.

"Total number of deaths 39—19 men and 20 women. The mortality has been low, being on the average number resident 9.22 per cent., and on the whole number treated 4.59 per cent. It will be perceived, from the above table, that many of those who died came to us in the last stages of serious organic disease. Of the eight deaths from phthisis, six were dying when admitted, two of them not able to sit up or speak, and were only sustained the short period of their residence by warmth and free stimulation. The seven cases reported as having died of exhaustion were wasted by disease or vicious habits. Three of them, at the development of the mania, had been purged, blistered, and profusely bled, and were brought to the asylum on beds. This injudicious treatment cannot be too strongly condemned. The recuperative powers were in these so far exhausted that no amount of care, stimulation and nutrition could arouse them. It may be proper to state that a number of persons received in a state of extreme feebleness, after long nursing, watchfulness, and free stimulation, recovered. The number of such cases received was 33. Seven of this number died, making 26 per cent. mortality in these cases.

